# Analysis of differential expression of tight junction proteins in cultured oral epithelial cells altered by *Porphyromonas gingivalis*, *Porphyromonas gingivalis* lipopolysaccharide, and extracellular adenosine triphosphate

**DOI:** 10.1038/ijos.2017.51

**Published:** 2018-03-10

**Authors:** Wei Guo, Peng Wang, Zhong-Hao Liu, Ping Ye

**Affiliations:** 1 Department of Endodontics, Yantai Stomatological Hospital, Binzhou Medical University, Yantai, China; 2 Department of Pediatrics, Yantai Stomatological Hospital, Binzhou Medical University, Yantai, China; 3 Department of Implant, Yantai Stomatological Hospital, Binzhou Medical University, Yantai, China; 4 Institute of Dental Research, Centre for Oral Health, Westmead Hospital, Westmead, Australia; 5 Faculty of Dentistry, the University of Sydney, Sydney, Australia

**Keywords:** junctional epithelium, periodontitis, *Porphyromonas gingivalis*, tight junctions

## Abstract

Tight junctions (TJs) are the most apical intercellular junctions of epithelial cells formed by occludin, claudins, junctional adhesion molecules (JAMs), and zonula occludens (ZO). Tight junction proteins can sense the presence of bacteria and regulate the transcription of target genes that encode effectors and regulators of the immune response. The aim of this study was to determine the impact of TJ proteins in response to *Porphyromonas gingivalis* (*P. gingivalis*), *P. gingivalis* lipopolysaccharide (*P. gingivalis* LPS), and extracellular adenosine triphosphate (ATP) in the oral epithelial cell culture model. Quantified real time-polymerase chain reaction (RT-PCR), immunoblots, and immunostaining were performed to assess the gene and protein expression in TJs. It was found that *P. gingivalis* infection led to transient upregulation of the genes encoding occludin, claudin-1, and claudin-4 but not JAM-A, claudin-15, or ZO-1, while *P. gingivalis* LPS increased claudin-1, claudin-15, and ZO-1 and decreased occludin, JAM-A, and claudin-4. Tight junction proteins showed significant upregulation in the above two groups when cells were pretreated with ATP for 3 h. The findings indicated that *P. gingivalis* induced the host defence responses at an early stage. *P. gingivalis* LPS exerted a more powerful stimulatory effect on the disruption of the epithelial barrier than *P. gingivalis*. ATP stimulation enhanced the reaction of TJ proteins to *P. gingivalis* invasion and LPS destruction of the epithelium.

## Introduction

Tight junctions (TJs), the most apical intercellular junctions of epithelial cells, are formed by occludin, claudins, junctional adhesion molecules (JAMs), and zonula occludens (ZO)-1, 2, and 3.^1^ Tight junctions are transmembrane proteins that control the paracellular passage and create a regulatable semipermeable diffusion barrier between individual cells.^2^ As the first integral membrane protein of TJs, occludin is the most ubiquitously expressed and the most reliable immunohistochemical marker for TJs.^3^ ZO-1 is critical to junction assembly.^4^ In the absence of ZO-1, cells fail to form TJs.^5^ JAM-A, another crucial transmembrane component of TJs, controls the passage of nutrients and solutes across epithelial surfaces^6^ and modulates many cellular functions, including cell migration, cell polarity, paracellular permeability, and proliferation.^7^ The claudin family is regarded as the backbone of TJs^8^ and contributes to the epithelial barrier in the junctional epithelium.^9^ The network of adhesion molecules in protein–protein interactions in gingival epithelial cells provides a clear picture for understanding the regulation of TJ proteins in periodontitis.^10^

*Porphyromonas gingivalis (P. gingivalis)*, a keystone pathogen of periodontitis colonizing the gingival sulcus, leads to the destruction of the soft tissues and bone that support the teeth.^11, 12^ Gingival epithelial cells function as a physical barrier against invading pathogens and have a significant role in host innate immune defences. However, the red-complex bacteria including *P. gingivalis* tend to suppress the innate immune responses of oral epithelial cells through different mechanisms to evade the host immune system, which results in persistent periodontal infection.^13^ Previously, we showed that *P. gingivalis* and its lipopolysaccharide (LPS) as a virulence factor dampened the end point innate immune responses by inhibiting the activation of the NLRP3 inflammasome.^14^ Furthermore, we demonstrated that extracellular adenosine triphosphate (ATP), a danger signal, resulted in the assembly of the NLRP3 inflammasome and secretion of mature cytokines in *P. gingivalis*-infected cells.^14^ Moreover, whether *P. gingivalis,* its LPS, and ATP can regulate the expression of the TJ proteins is unknown. The aim of this study was to determine the impact of TJ proteins in response to *P. gingivalis*, *P. gingivalis* LPS, and extracellular ATP in the oral epithelial cell culture model, and their effects were enhanced by pre-exposure of epithelial cells to extracellular ATP molecules.

## Materials and methods

### Oral epithelial cell culture

The epithelial cell line H413 was used in this study. The H413 cell line was derived from a human oral squamous cell carcinoma, and it displays stratified epithelial cell morphology in culture^15^ H413 clonal cell lines were established using a limited dilution method as described previously.^16^ H413 clone-1 cells were cultured in Eagle’s minimum essential medium (JMEM, Joklik modification, Sigma-Aldrich, St Louis, MO, USA) containing supplements including penicillin/streptomycin (100 IU·mL^−1^, Sigma) and 10% foetal calf serum (FCS, CSL, Victoria, Australia) at 37 °C in 5% CO_2_.^17^ Cells were subcultured every 3 days; they were first collected using TrypLE Express (trypsin replacement, Invitrogen, Life Technologies, Carlsbad, CA, USA) in PBS and then placed into fresh Eagle’s medium.

### Bacterial cell culture

*P. gingivalis* (ATCC 33277) was used in this study. *P. gingivalis* was cultured in trypticase soy broth supplemented with haemin (5 mg·mL^−1^, Sigma) and menadione (1 mg·mL^−1^, Sigma) at 37 °C under anaerobic conditions for 24 h. Prior to treatments of H413 clone-1 cells, the bacteria were centrifuged at 5 000 r·min^-1^. and 4 °C for 15 min, washed twice, and re-suspended in cold PBS, pH 7.3.

### Cell treatment

Confluent H413 clone-1 cell cultures (5 × 10^6^ cells in T-25 cm^2^ flasks) were washed three times with PBS and then incubated with either *P. gingivalis* at a multiplicity of infection of 100 or ultrapure LPS from *P. gingivalis* at the concentration of 1 μg·mL^−1^ (InvivoGen, San Diego, CA, USA) in cell culture media for 2 and 4 h.^18^ H413 clone-1 cells without any treatment were used as the negative control.

Experiments were also performed after pre-incubation of H413 clone-1 cells with 5 mM ATP (InvivoGen) for 3 h prior to incubation with *P. gingivalis* or LPS-Pg for 2 and 4 h. For these experiments, H413 clone-1 cells pre-incubated with 5 mmol·L^-1^ ATP for 3 h were used as the negative control.

### RNA isolation and quantitative real-time polymerase chain reaction(PT-PCR)

After treatments, H413 clone-1 cells were collected in 1 mL of Trizol reagent (Invitrogen), and RNA was extracted from each sample following the manufacturer’s instructions. Complementary DNAs (cDNAs) were synthesized using SuperScript III Reverse Transcriptase (Invitrogen) according to the manufacturer’s protocol employing the oligo (dT)_12-18_ primer. The cDNA samples were subjected to quantification of TJ proteins by RT-PCR.

The sequences of the PCR primers used for amplification of occludin, JAM-A, claudin-1, claudin-4, claudin-15, and ZO-1 were designed using Oligo Explorer software (1.1.0) and synthesized by Integrated DNA Technologies (IDT, Coralville, IA, USA).^19^ Real-time polymerase chain reaction analyses were performed with SYBR Green using the Stratagene MxPro-Mx3005P System (Agilent Technologies, Santa Clara, CA, USA). Polymerase chain reactions (20 μL) contained cDNA (2 μL), 200 nmol.L^-1^ of each forward and reverse primer, and Platinum SYBR Green qPCR SuperMix-UDG (Invitrogen). Complementary DNA samples isolated from H413 clone-1 cells without treatment were quantified by a PicoGreen kit (Invitrogen) to construct standard curves (2-2 000 pg) by reference to the expression of the housekeeping gene encoding β-actin. The thermal cycling conditions were 95 °C for 2 min followed by 40 cycles of 95 °C for 15 s and 60 °C for 30 s. The results were analysed using MxPro 4.10 software. All experiments were performed in triplicate wells and repeated at least three times.

### Detection of occludin, JAM-A, claudin-1, claudin-4, claudin-15, and ZO-1 proteins by western blot

H413 clone-1 cells were treated with different conditions as described above. Whole cell proteins were prepared by scraping the cells in cold PBS, extracted in SDS sample buffer, separated by SDS-PAGE using 5% to 12% gradient mini-gels, transferred to nitrocellulose membranes (Bio-Rad), and blocked overnight with 3% BSA (Sigma) in 0.1 mol·L^-1^ Tris buffered salts solution pH 7.4 (TBS). The nitrocellulose membranes were then incubated with rabbit polyclonal antibodies to human occludin (ab31721), JAM-A (ab106114), claudin-1 (ab15098), claudin-4 (ab53156), ZO-1 (ab59720), claudin-15 (ab215354; all 1 μg·mL^−1^, Abcam, Cambridge, UK), and β-actin (0.1 μg·mL^−1^, GenTex, Zeeland, MI, USA) in 0.05% Tween20/TBS for 4 h. β-actin was used as a loading control. After the incubation, the membranes were washed three times and subsequently incubated with alkaline phosphatase (AP)-conjugated secondary antibody (goat-anti rabbit IgG, DAKO) diluted 1:1 500 in Tween20/TBS for 2 h. Bound antibodies were displayed with AP substrate (Bio-Rad) after the development of reactivity for proteins.

### Immunostaining and confocal laser scanning microscopy

Confluent H413 clone-1 cells (2 × 10^5^ per cm^2^) in eight-well chamber slides (Corning) were incubated overnight at 4 °C with primary antibodies: rabbit anti-human occludin, JAM-A, claudin-1, claudin-4, ZO-1, claudin-15 (5 μg·mL^−1^, Invitrogen), in 10% FCS/PBS at room temperature for 1 h in a humid chamber. After washing with PBS, the goat anti-rabbit IgG Alexa Fluor 488 secondary antibody (Invitrogen) was added for 1 h at room temperature. The primary antibody was replaced with control rabbit Ig (DAKO) as the negative control. Wells were washed with PBS three times and mounted onto glass slides using ProLong Gold antifade reagent with DAPI (Molecular Probes, Invitrogen).

Confocal images were acquired with an Olympus Fluoview (FV) 1000 system equipped with Olympus FV 10-MCPSU (405, 473, and 633 nm) and NTT electronic Optiλ (559 nm) lasers. All fluorescence images prepared with confocal acquisition software (FV10-ASW 1.7) were stored and exported as TIF files.

### Statistical analysis

All data were analysed by an analysis of variance test from at least three consecutive experiments for RT-PCR. *P*<0.05 was considered statistically significant.

## Results

### Expression of genes encoding the tight junction proteins occludin, JAM-A, claudin-1, claudin-4, claudin-15, and ZO-1 in response to *P. gingivalis*, *P. gingivalis* LPS, extracellular adenosine triphosphate (ATP) plus *P. gingivalis*, and ATP plus *P. gingivalis* LPS

The expression of six genes encoding TJ proteins was examined in this study. In all performed experiments, there were no significant changes in gene expression between unstimulated control groups and control groups pretreated with ATP for 3 h.

**Figure 1:**
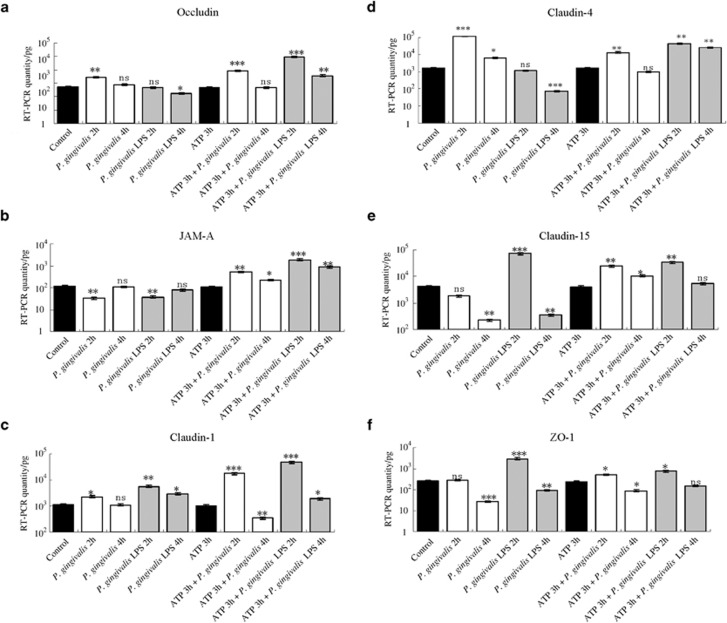
**Tight junctions’ gene expressions.** Gene expressions of occludin (**a**), JAM-A (**b**), claudin-1 (**c**), claudin-4 (**d**), claudin-15 (**e**), and ZO-1(**f**). Significant changes in gene expressions altered by different treatments with *P. gingivalis* infection (2 and 4 h), *P. gingivalis* LPS stimulation (2 and 4 h), and ATP plus *P. gingivalis* or *P. gingivalis* LPS (2 and 4 h) in H413 clone-1 epithelial cells. *P<0.05, **P<0.01, ***P<0.001, ANOVA test. ANOVA, analysis of variance; ATP, adenosine triphosphate; JAM, junctional adhesion molecule; LPS, lipopolysaccharide; ZO, zonula occludens.

**Figure 2:**
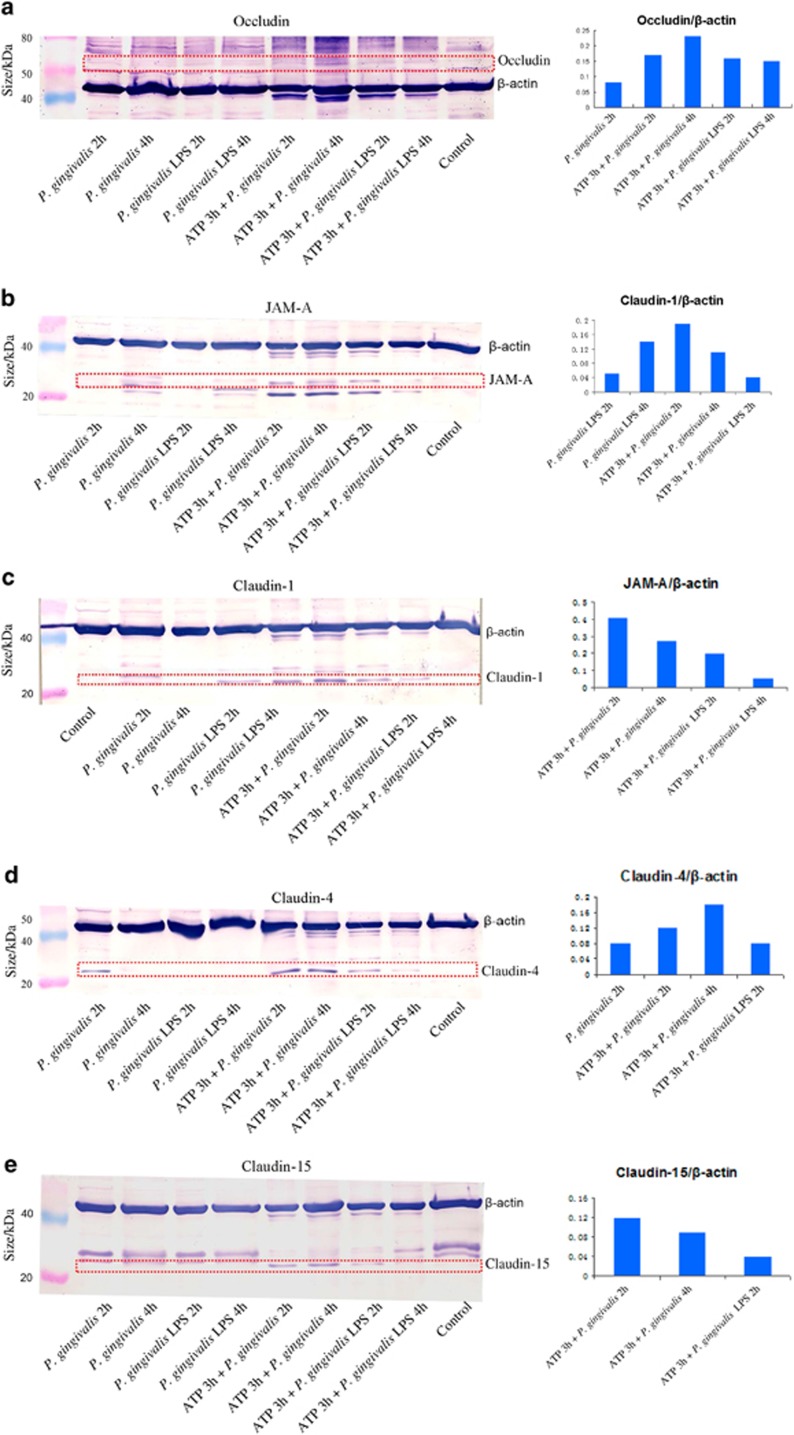
**Tight junctions' protein expressions.** Western blots showing occludin (**a**), JAM-A (**b**), claudin-1 (**c**), claudin-4 (**d**), claudin-15 (**e**) protein bands corresponding to their gene expression in H413 clone-1 epithelial cells in response to *P. gingivalis*, *P. gingivalis* LPS, and ATP plus *P. gingivalis* or *P. gingivalis* LPS. ATP, adenosine triphosphate; JAM, junctional adhesion molecule; LPS, lipopolysaccharide.

As shown in Figure 1a, there was a significant upregulation of occludin gene expression after *P. gingivalis* infection for 2 h and downregulation after *P. gingivalis* LPS stimulation for 4 h compared with the unstimulated control group (*P*<0.05). Pretreatment with ATP for 3 h enhanced the occludin gene expression induced by *P. gingivalis* for 2 h (*P*<0.01) or by *P. gingivalis* LPS (2 and 4 h). This indicated that the activation of occludin depended on ATP. At the protein level, the proteolysis of occludin corresponded to the gene expression with ATP pretreatment (Figure 2a).

Figure 1b shows that there was downregulation of JAM-A messenger RNA (mRNA) levels after infection with *P. gingivalis* or *P. gingivalis* LPS for 2 h compared with JAM-A levels in the control group. However, when cells were pre-treated with ATP for 3 h, we found markedly increased JAM-A gene expression under treatment with *P. gingivalis* and *P. gingivalis* LPS at 2 h and then slightly reduced expression at 4 h in both groups. At the protein level (Figure 2b), when cells were pretreated with ATP for 3 h and then incubated with *P. gingivalis* and *P. gingivalis* LPS for 2 h and 4 h, strong bands were observed for JAM-A that corresponded to gene expression. This finding suggested that *P. gingivalis and P. gingivalis* LPS rely on ATP for activation of JAM-A.

Figure 1c shows that significant increases in claudin-1 mRNA levels were found after infection with *P. gingivalis* for 2 h and stimulation with *P. gingivalis* LPS for 2 and 4 h compared with the levels in the control group. Interestingly, for ATP plus *P. gingivalis* infection, marked upregulation at 2 h and downregulation at 4 h were observed compared with that in the ATP pretreatment group. For ATP plus *P. gingivalis* LPS, there was a pattern similar to that seen with ATP plus *P. gingivalis* infection, but all were upregulated. At the protein level (Figure 2c), changes similar to those with gene expression in claudin-1 were observed, which indicated that *P. gingivalis* and *P. gingivalis* LPS both had the ability to up-regulate the expression of claudin-1, and this effect was stronger after pre-incubation with ATP.

Figure 1d shows that during the course of *P. gingivalis* infection, there were significant increases in claudin-4 gene expression (2 and 4 h) compared with the control group, and significant decreases in claudin-4 gene expression were observed at 4 h with stimulation by *P. gingivalis* LPS. After cells were pre-incubated with ATP for 3 h and then infected with *P. gingivalis* or stimulated with *P. gingivalis* LPS, there was upregulation of claudin-4 at 2 h or 2 and 4 h, respectively, compared with levels in the ATP pretreatment group. At the protein level (Figure 2d), trends in the proteolysis of claudin-4 bands corresponded to that of gene expression. This showed that the activation of claudin-4 depends on *P. gingivalis* and ATP, but not *P. gingivalis* LPS.

As evident in Figure 1e, the data showed the obvious reduction in claudin-15 gene expression in *P. gingivalis*-infected group at 4 h and the significant increase in the *P. gingivalis* LPS-stimulated group at 2 h followed by a steep decrease at 4 h. After cells were pre-incubated with ATP for 3 h, the claudin-15 mRNA levels in both the *P. gingivalis* infection (2 and 4 h), and *P. gingivalis* LPS stimulation (2 h) groups were significantly upregulated when compared with the ATP pretreatment group. At the protein level, claudin-15 levels corresponded to gene expression except for the cells treated with *P. gingivalis* LPS for 2 h (Figure 2e). These results indicated that claudin-15 activation relied on *P. gingivalis* LPS and ATP.

As shown in Figure 1f, during the infection with *P. gingivalis*, there were significant decreases in ZO-1 mRNA levels at 4 h compared with those in the control group; in contrast, a remarkable increase in the ZO-1 mRNA level was observed after 2 h of stimulation with *P. gingivalis* LPS. After ATP pretreatment for 3 h, ZO-1 levels were observed to be upregulated at 2 h and downregulated at 4 h with *P. gingivalis* infection and upregulated at 2 h with *P. gingivalis* LPS stimulation compared with the ATP pretreatment groups. There were no obvious changes detected in ZO-1 protein level by immunoblots (data not shown).

### Immunostaining of occludin, JAM-A, claudin-1, claudin-4, claudin-15, and ZO-1 in response to *P. gingivalis*, *P. gingivalis* LPS, ATP plus *P. gingivalis*, and ATP plus *P. gingivalis* LPS in epithelial cells

**Figure 3:**
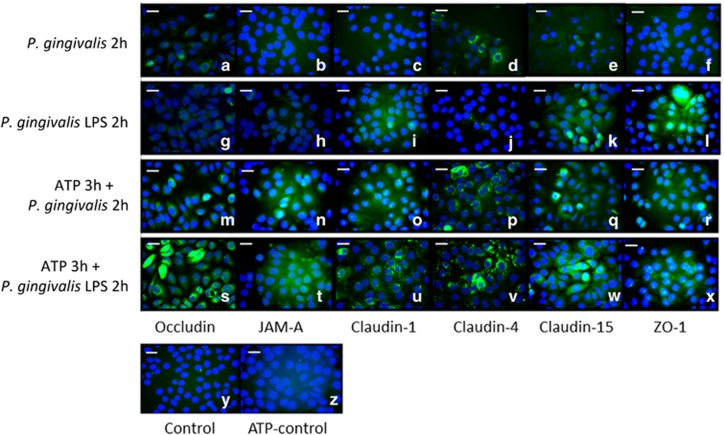
**Tight junctions' staining.** Occludin, JAM-A, claudin-1, claudin-4, claudin-15, and ZO-1 staining (**a**–**x**): H413 clone-1 cells treated with *P. gingivalis* infection (**a**–**f**), *P. gingivalis* LPS stimulation (**g**–**l**), and ATP plus *P. gingivalis* (**m**–**r**), or *P. gingivalis* LPS (**s**–**x**) for 2 h. Only moderate expression was observed for claudin-4 (**d**) after *P. gingivalis* infection; weak staining was detected for claudin-1 (**i**), and the majority of immune-reactivity was detected for claudin-15 (**k**) and ZO-1 (**l**) with *P. gingivalis* LPS treatment. After ATP pretreatment for 3 h, significant staining for occludin, JAM-A, claudin-1, claudin-4, claudin-15, and ZO-1 were observed in both groups (**m**–**x**), control (y), ATP-control (z), error bar, 20 μm. There were similar patterns with different treatments for 4 h (data not shown). ATP, adenosine triphosphate; JAM, junctional adhesion molecule; LPS, lipopolysaccharide; ZO, zonula occludens.

Immunostaining was used to evaluate TJ protein expression. During *P. gingivalis* infection, weak expression of occludin and moderate expression of claudin-4 were observed (Figure 3a and 3d). The cells were negative for JAM-A, claudin-1, claudin-15, and ZO-1 (Figure 3b, 3c, 3e, and 3f). After *P. gingivalis* LPS stimulation, weak staining was shown for claudin-1 (Figure 3i), and the majority of immune-reactivity was detected for claudin-15 (Figure 3k) and ZO-1 (Figure 3l). After ATP pretreatment for 3 h, increased expression of occludin, JAM-A, claudin-1, claudin-4, claudin-15, and ZO-1 were observed in both the *P. gingivalis* infection group and the *P. gingivalis* LPS stimulation group (Figure 3m–3x) compared with the negative control and ATP pretreatment control (Figure 3y and 3z). These comparisons indicated significant differences after ATP pretreatment.

## Discussion

The present study demonstrated the reaction of a H413 clone-1 epithelial cell model to the primary oral pathogen *P. gingivalis*, *P. gingivalis* LPS, and ATP. The cell model used in this study has been confirmed to show a typical epithelial morphology and high expression of CD24 (a cancer marker) to mimic the important periodontal feature of the epithelial attachment to the tooth and diseased epithelial lining of periodontitis.^20, 21^ Hence, this cell model can produce consistent and reproducible findings compared to primary epithelial cells, which generally have a very limited lifespan (some only four passages) and are slow to proliferate.

Periodontitis infection is caused by the formation of subgingival biofilms on the surface of the tooth. A recent study has shown that biofilms generated *in vitro* can affect the selected desmosomal junction expression in gingival epithelial cells, with the potential involvement of the “red complex” species, including *P. gingivalis*; however, there was a limited effect of the biofilms on the expression of tight, adherens, and gap junctions. This could compromise the structural integrity of the gingival tissue, favouring individual bacterial invasion.^22^ On the proteomic level, secreted proteins were downregulated in the “red complex” biofilm-challenged gingival epithelium. This downregulation may dampen the host early innate immune responses in order for the individual bacteria of species such as *P. gingivalis* to evade elimination and survival longer.^23, 24, 25^ *P. gingivalis* is a major aetiologic agent in the “red complex” biofilm that leads to slow but steady disruption of the supporting structures of the teeth. The results of the present study indicate that cell infection with *P. gingivalis* promoted the expression of the TJ genes encoding occludin, claudin-1 and claudin-4, which enhances barrier function and decreases cell-cell permeability. Occludin knockout mice are viable but fail to display effective barrier function.^26^ Claudin-1 is a 23-kDa integral membrane protein that is identified second in TJ strands.^27^ Knockout of claudin-1 in mice leads to loss of the tight junctional barrier to water and macromolecules at the stratum granulosum of the epidermis.^28^ Claudin-4 exhibited immunoreactivity in the intercellular spaces of all layers of the oral epithelium and the junctional epithelium. Downregulation of claudin-4 causes increased permeability.^29^

In a normoxic incubator, because cells absorb oxygen from the medium, there is an oxygen gradient from the top gas phase (20%), down to the medium phase (8%–18%), the pericellular phase (1%–7.5%), and the bottom of the flask (0).^30, 31^ Oxygen exchange can be affected by many factors, such as the efficiency of air circulation in the incubator, tightness of the sealed caps and whether or not filter caps used, culture age, medium depth and cell density. A longer culture time (4 days) and confluent cells, which were the conditions used in our study, creates a lower oxygen level. Lewis *et al.*^32^ revealed that *P. gingivalis* could grow nearly as well under anaerobic and microaerophilic (6% oxygen) conditions. Accordingly, there are no effects or differences after 2 h or 4 h of *P. gingivalis* infection under aerobic cell culture conditions. Nevertheless, *P. gingivalis* is able to permeate and survive within the gingival epithelial cells despite not being contained in an anaerobic environment for up to 48 h.^33, 34^

*P. gingivalis* has developed sophisticated strategies aimed at inducing host defence responses during the first stage and then perturbing the structural and functional integrity of the junctional epithelium that results in local tissue destruction,^35^ a mechanism that may significantly contribute to the initiation of pocket formation and attachment loss.^35^

*P. gingivalis* LPS, as an important virulence factor eliciting the inflammatory response in periodontal disease^36^ exerts a powerful stimulatory effect on the destruction of the epithelial barrier because of the increase in cell–cell polarity and permeability to degrade junctional complexes, and it is more overtly, specifically and directly aggressive than *P. gingivalis.*^35, 36^ However, in the early stage of *P. gingivalis* LPS infection, we found that *P. gingivalis* LPS increased the gene expression of claudin-1, claudin-15, and ZO-1 and decreased the gene expression of occludin, JAM-A, and claudin-4. Knockout of claudin-15 in mice causes congenital enlargement of the small intestine.^37^ ZO-1 is essential to junction assembly.^4^ JAM-A is crucial to cell–cell polarity.^38^

ATP, an external signal from dying or injured cells, contributes to more complex mechanisms of increased bacterial invasion and disease progression by enhancing the reaction of TJs and bacteria, and significantly, these interactions are central to the development of periodontal disease and in particular chronic periodontitis.^39^ However, at the early stage, our findings indicate that pretreatment with ATP for 3 h facilitates the expression and secretion of TJ proteins against infection with *P. gingivalis* and its LPS. This is because extracellular ATP results in assembly of an inflammasome NLRP3, activation of caspase-1, and secretion of mature cytokine interleukin (IL)-1β. IL-1β was secreted when LPS-treated or *P. gingivalis*-infected cells were subsequently stimulated with ATP.^40^ The NLRP3 inflammasome acts an essential mediator (good and bad)^41^ of the inflammatory response in the gingival epithelium.^40^

## Conclusion

Chronic periodontitis presents increased bacterial invasion, altered TJ protein expression, and a sophisticated immune defence response. TJ strands represent multiprotein complexes of transmembrane proteins. The various positive or negative correlations shown in this study do not prove a causal relationship, and the precise role of each factor in the regulation of TJ protein expression warrants further investigation. These findings may provide some insights into the host responses at the initial stages of infection.
